# Impacts of Phantom Off-Center Positioning on CT Numbers and Dose Index CTDIv: An Evaluation of Two CT Scanners from GE

**DOI:** 10.3390/jimaging7110235

**Published:** 2021-11-10

**Authors:** Xiaoming Zheng, Lachlan Gutsche, Yazan Al-Hayek, Johanna Stanton, Wiam Elshami, Kelsey Jensen

**Affiliations:** 1 Medical Radiation Science, School of Dentistry and Medical Sciences, Faculty of Science and Health, Charles Sturt University, Wagga Wagga, NSW 2678, Australia; lachlangutsche7@gmail.com (L.G.); yal-hayek@csu.edu.au (Y.A.-H.); 2 I-Med Radiology Network, 36 Hardy Ave, Wagga Wagga, NSW 2650, Australia; Johanna.Stanton@i-med.com.au; 3 Department of Diagnostic Imaging, University of Sharjah, Sharjah 27272, United Arab Emirates; welshami@sharjah.ac.ae; 4 Veterinary Clinical Center, School of Agricultural Environmental and Veterinary Sciences, Faculty of Science and Health, Charles Sturt University, Wagga Wagga, NSW 2678, Australia; kejensen@csu.edu.au

**Keywords:** dose CTDIv, CT numbers, off-center positioning, scan projection radiograph

## Abstract

The purpose of this work is to evaluate the impacts of body off-center positioning on CT numbers and dose index CTDIv of two scanners from GE. HD750 and APEX scanners were used to acquire a PBU60 phantom of Kagaku and a 062M phantom of CIRS respectively. CT images were acquired at various off-center positions under automatic tube current modulation using various peak voltages. CTDIv were recorded for each of the acquisitions. An abdomen section of the PBU60 phantom was used for CT number analysis and tissue inserts of the 062M phantom were filled with water balloons to mimic the human abdomen. CT numbers of central regions of interests were averaged using the Fiji software. As phantoms were lifted above the iso-center, both CTDIv and CT numbers were increased for the HD750 scanner whilst they were approximately constant for the APEX scanner. The measured sizes of anterior-posterior projection images were also increased for both scanners whilst the sizes of lateral projection images were increased for the HD750 scanner but decreased for the APEX scanner. Off-center correction algorithms were implemented in the APEX scanner. Matching the X-ray projection center with the system’s iso-center could improve the accuracy of CT imaging.

## 1. Introduction

Patient vertical off-center positioning is common in clinical CT imaging [1,2]. It can cause an increased surface dose to patients [1] and varied CT numbers at different locations within the bodies of patients [2]. Computer software may be used to automatically adjust patient’s positions during CT scans [3,4] or auto couch height position compensation can be applied to correct for the mis-positioning and reduce radiation doses to patients [5]. More recently, optical camera technologies have also been used to improve patient positioning during CT imaging [6,7]. Not all CT scanners currently being used in clinical practice have off-center correction algorithms implemented [8,9]. Radiologic technologists can only guess if an off-center correction algorithm is implemented in a clinical scanner. The correction algorithms themselves are proprietary information of the manufacturers [5,9].

Automatic tube current modulation (ATCM) has long been implemented in clinical CT scanners by all manufacturers to minimize radiation doses to patients whilst maintaining diagnostic image quality [9]. Different manufacturers may use different image quality indexes in guiding tube current modulation, such as the image noise index (NI) used by the manufacturer GE. All manufacturers use scan projection radiographs (SPR) or localizers to estimate the body sizes of patients so that the tube current can be adjusted according to the thickness of the patient’s body, i.e., a higher current is applied where body’s diameter is thicker. As the human body’s shape is elliptical, measurements of the two major diameters of an ellipse are required [10]. Two SPRs are generally acquired in practice: anterior-posterior (AP) or posterior-anterior (PA) and lateral (LAT) projections. The difference between PA and AP projection is that there is a patient table attenuation before or after the attenuation of patients. The resulting effect is that patients receive a higher dose from an AP radiograph in comparison with that of a PA radiograph [11] because the patient table attenuates more X-ray photons in PA projection than that in AP radiograph, assuming that the same number of photons arrived at the detectors. ATCM takes the reduced photon flux through the body of a patient as a thicker body, and therefore applies a higher tube current. As a result, PA localizer has a higher CTDIv than that of an AP localizer in ATCM [12,13,14,15,16]. The American Association of Physicists in Medicine (AAPM) recommends AP and LAT projection images be used for the calculations of body sizes [10]. In general, the patient’s body size can be estimated by using a single SPR projection, either AP or PA or LAT, assuming an elliptical shape of human body [17,18]. Manufacturers, therefore, provide options of selecting one of the three projections for the ATCM. These settings are satisfactory if the patients are positioned at the system’s iso-center. Problems arise when the patient is not perfectly positioned at the iso-center, or off-center positioning, which is often the case in clinical practice [1,2]. Off-center positioning means that patient size in the SPR is either magnified or minified when the patient is off-centered or closer to the X-ray tube depending on AP or PA projections. If the SPR is used to estimate the body size for the ATCM without correction, the applied tube current will be either excessive or inadequate and so will the dose to patients.

CT numbers in Hounsfield units (HU) were also found to be varied under off-center positions [2,19]. The variation of CT numbers is more complicated as bowtie filters are generally installed in CT scanners to compensate for the elliptical shape of the human body, i.e., a higher body’s attenuation at the center and a lower body’s attenuation at the peripheral. The purpose of the bowtie filters is to make a uniform photon intensity at the detectors. The X-ray beam that passes through the bowtie filter at its peripheral becomes harder than those photons passed through the bowtie center because they experience a thicker peripheral bowtie attenuation. The difference in effective energy from beam hardening could be up to 10 kV [19]. For an off-center position, the X-ray beam attenuation is a combination of the bowtie filters and the body’s thickness which is dependent on the projection angles [20]. For a center region of the body, the combined attenuation of the bowtie and body’s thickness varies approximately sinusoidal as the projection angle changes [20]. The variation of the combined body size and bowtie attenuation can be very different at different regions within the body. It should be noted that the variation of dose is proportional to the variation of tube current in ATCM whilst CT number or attenuation coefficient is dependent on effective beam energy or kVp but independent of the tube current or mAs.

The purposes of this work are to (i) measure the impacts of the off-center positioning on dose index CTDIv and CT numbers in Hounsfield units using two models of CT scanners from the same manufacturer (GE); (ii) provide practicing radiologic technologists with updated information on the implementation of any off-center correction algorithms in clinical CT scanners; and (iii) provide guidance on selecting the best imaging parameters for the benefits of patients.

## 2. Materials and Methods

**Figure 1:**
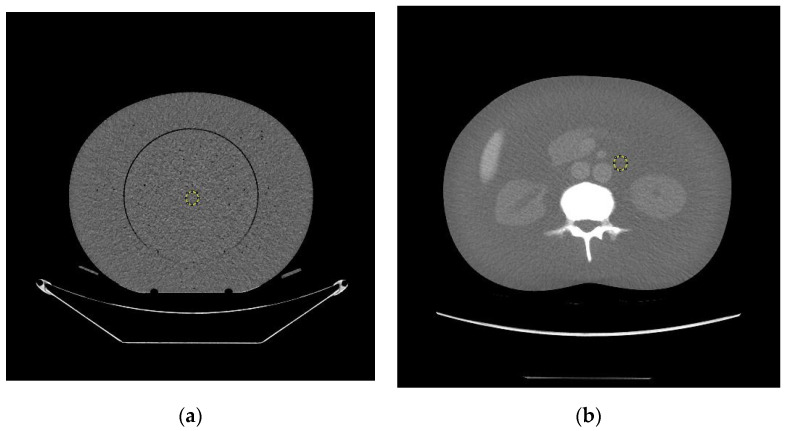
The central slices selected for CT number measurements of (**a**) the 062M electron density phantom; (**b**) the PBU60 anthropomorphic phantom. The central regions of interest used in this study are shown as yellow circles. The horizontal axis is termed body width and the vertical axis is termed body thickness. Both data collection and image reconstruction are 50 cm in diameter for both phantoms.

A Revolution APEX and a Discovery HD750 CT scanner of General Electric (GE) were used to acquire images of an electron density phantom (model 062M, 27 cm × 33 cm) of computerized imaging reference systems (CIRS), Inc. (Norfolk, VA, USA) and an anthropomorphic phantom (model PBU-60, 165 cm in length and 50 kg in weight) of Kyoto Kagaku Co. (Kyoto, Japan), respectively. All tissue insert holes within the electron density phantom were filled with water balloons to mimic an abdomen section of human body [21]. Both phantom images were acquired under automatic tube current modulation (ATCM) mode using default noise index and under body (abdomen) bowtie filters. Four kVps of 80, 100, 120 and 140 were applied to the image acquisitions of the electron density phantom 062M using the Revolution APEX scanner and three kVps of 80, 120 and 140 were selected in acquiring the PBU-60 anthropomorphic phantom using the Discovery HD750 scanner. All images were reconstructed in slice thicknesses of 5 mm. Images of the PBU-60 phantom were acquired at vertical off-center positions of 0 (iso-center), ±3, ±6, ±10 cm from the iso-center using the HD750 scanner whilst images of the 062M phantom were acquired at vertical 0 (iso-center), 3, 6, 9 cm above the iso-center. Both AP and PA as well as LAT localizers (SPRs) were acquired for the PBU-60 phantom whilst AP and LAT SPRs were acquired for the 062M electron density phantom. The projection image sizes (horizontal width and vertical thickness as defined in Figure 1) were measured directly on the SPRs. The values of CT dose index CTDIv were extracted from the dose report files (in DICOM format) of each acquisition for all off-center positions and for both phantoms and CT scanners. The CTDIv is an indication of scanner’s dose output given a specific set of imaging parameters such as kVp and mAs. A size-dependent correction factor needs be applied to the CTDIv in estimating the dose delivered to a specific patient [22]. CT numbers (in HU) were determined by drawing a central region of interest (ROI) and averaging 5 central slices of the 062M phantom and 5 slices from the abdomen section of the PBU-60 phantom as shown in Figure 1. The Fiji (ImageJ) software [23] was used to average the CT numbers and measure the width and thickness of the scan projection radiographs (SPRs).

## 3. Results

**Figure 2:**
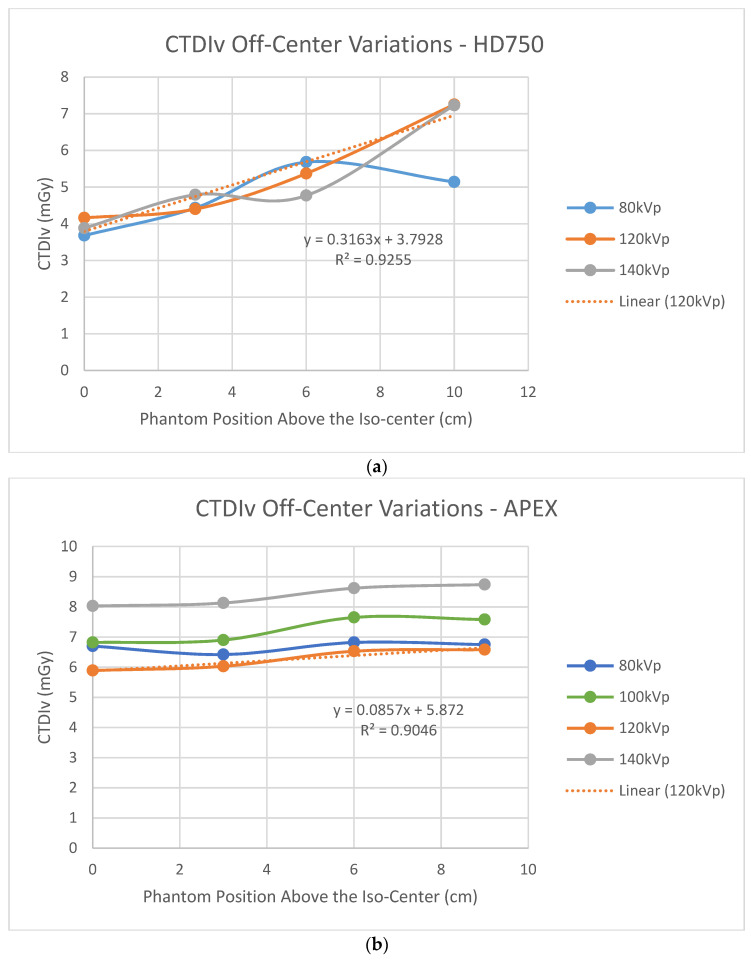
Off-center variations of dose index CTDIv; (**a**) the Discovery HD750 scanner using the PBU60 phantom; (**b**) the Revolution APEX scanner using the 062M phantom.

Figure 2 shows dose index CTDIv variations at the phantom’s various off-center positions above the system’s iso-center. Figure 2a shows that CTDIv increased as the height of the phantom above the iso-center increased for the HD750 scanner whilst CTDIv was approximately constant as the phantom was lifted above the iso-center for the APEX scanner. Figure 2a is consistent with the result of Paolicchi et al. [24] on a HD750 scanner. Figure 2b is consistent with the measured results of a Philips’ iCT256 scanner by Paolicchi et al. [24].

**Figure 3:**
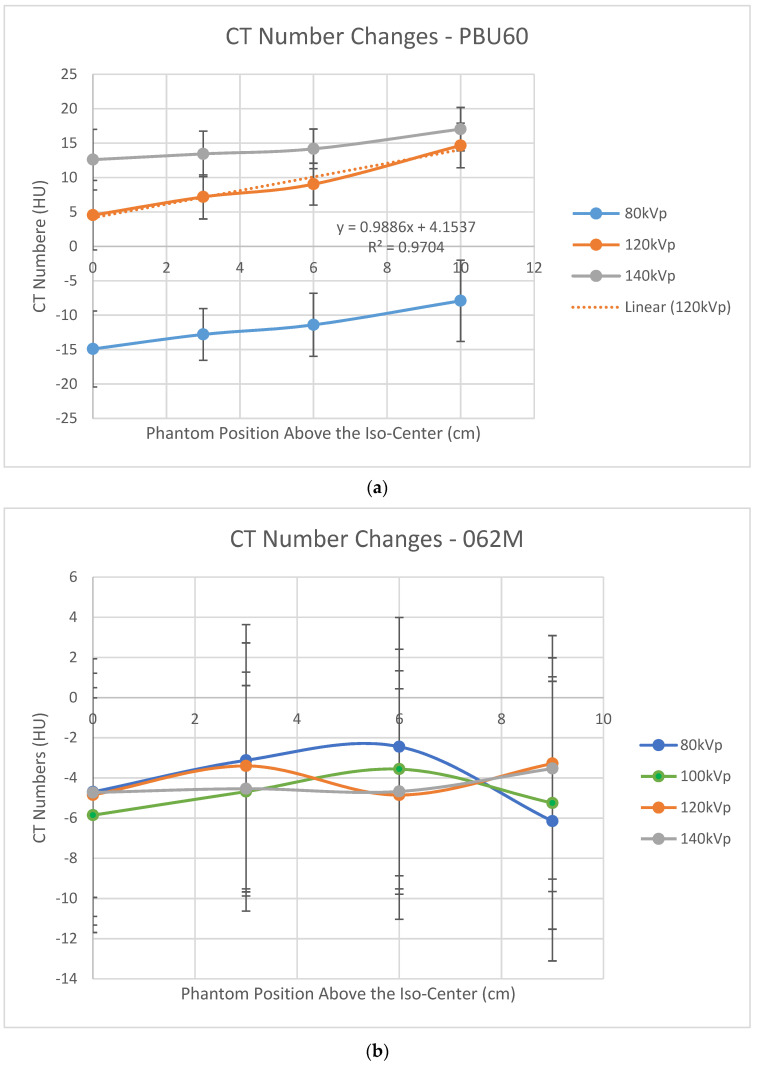
CT number variations as phantoms are lifted above the iso-center; (**a**) the PBU60 phantom using the HD750 scanner; (**b**) the 062M phantom using the APEX scanner.

Figure 3a shows that CT numbers at the central region of the PBU60 phantom increased as the phantom was lifted above the iso-center using the HD750 scanner. This result is consistent with that of a water phantom using a vintage GE scanner [19]. In contrast, the variations of CT numbers at the center of the 062M phantom appear sinusoidal within a small range of ±2 HU using the APEX scanner as shown in Figure 3b.

**Figure 4:**
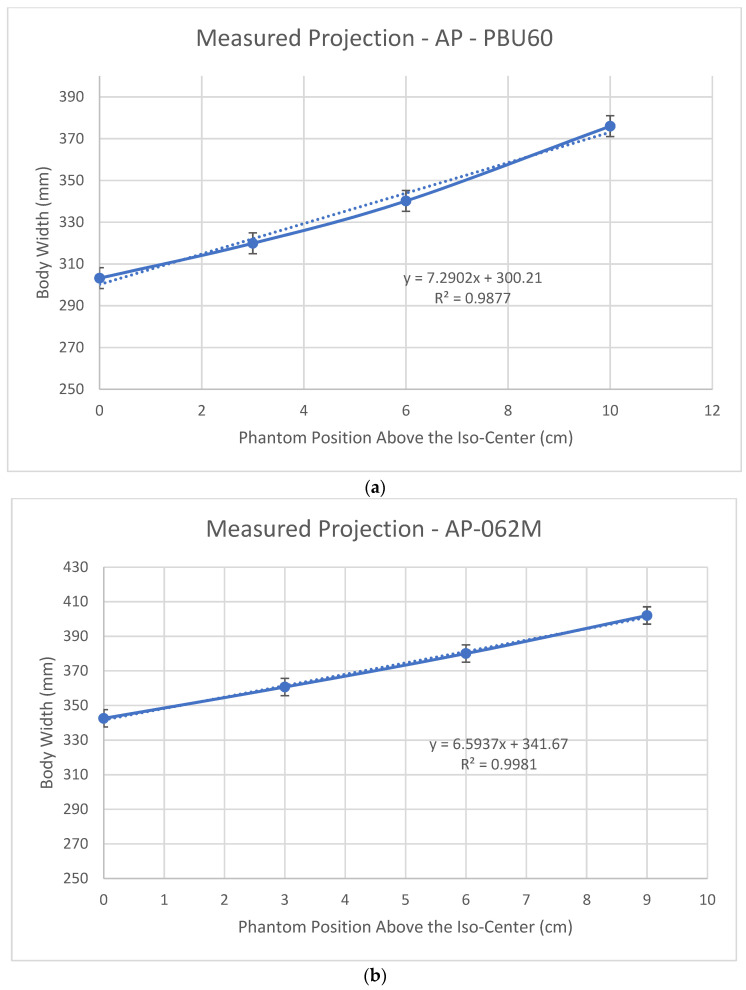
Measured body widths from the SPR images using an AP localizer: (**a**) the PBU60 phantom using the HD750 scanner; (**b**) the 062M phantom using the APEX scanner.

Figure 4 shows the body widths (defined in Figure 1) measured from the SPR images using an AP localizer for the PBU60 phantom using the HD750 scanner and 062M phantom using the APEX scanner. It shows a linear increased (magnified) body width as the phantoms are lifted above the iso-center for both scanners. This linear relationship agrees with the results of Matsubara et al. [25]. The rate of size increase (the slope) of the HD750 is slightly higher than that of the APEX scanner (slopes 7.29 vs. 6.59 as shown in Figure 4).

**Figure 5:**
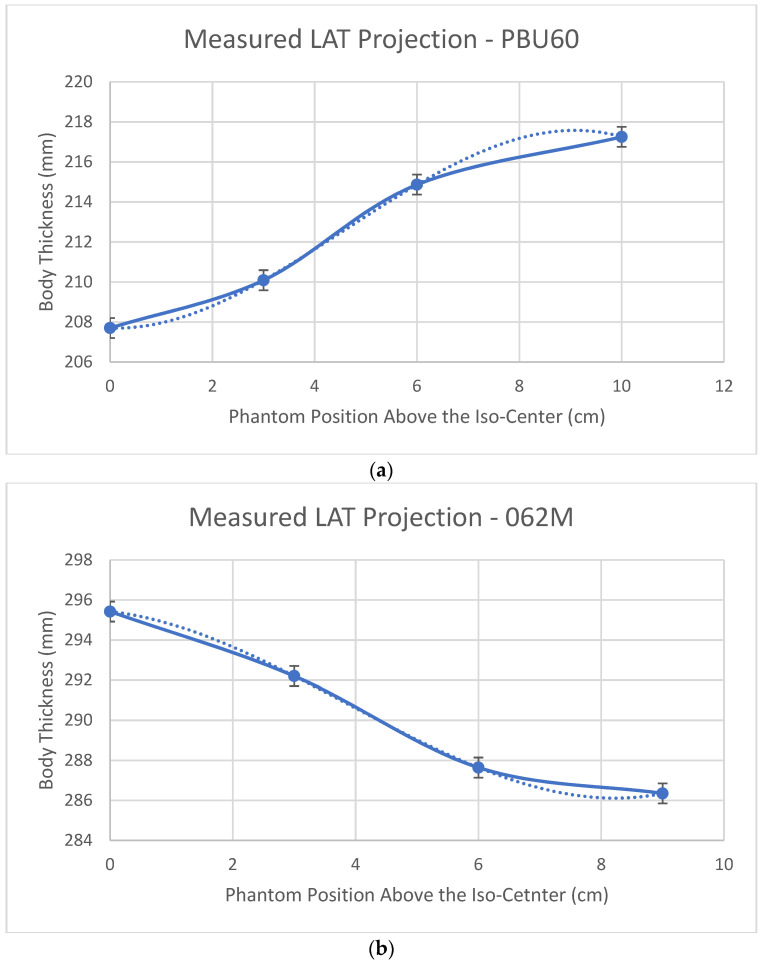
Body thickness measured from the SPR images using a LAT localizer: (**a**) the PBU60 phantom using the HD750 scanner and (**b**) the 062M phantom using the APEX scanner.

Figure 5 shows body thickness (defined in Figure 1) measured from LAT SPR images of the PBU60 phantom using the HD750 scanner and 062M phantom using the APEX scanner. The difference between HD750 and APEX scanners is that body thickness is magnified with the HD750 scanner whilst minified with the APEX scanner, although the magnitudes of the magnification or minification are relatively small in comparison with SPR images using the AP localizer (±1 cm vs. 10 cm at a 10 cm off center position).

**Figure 6:**
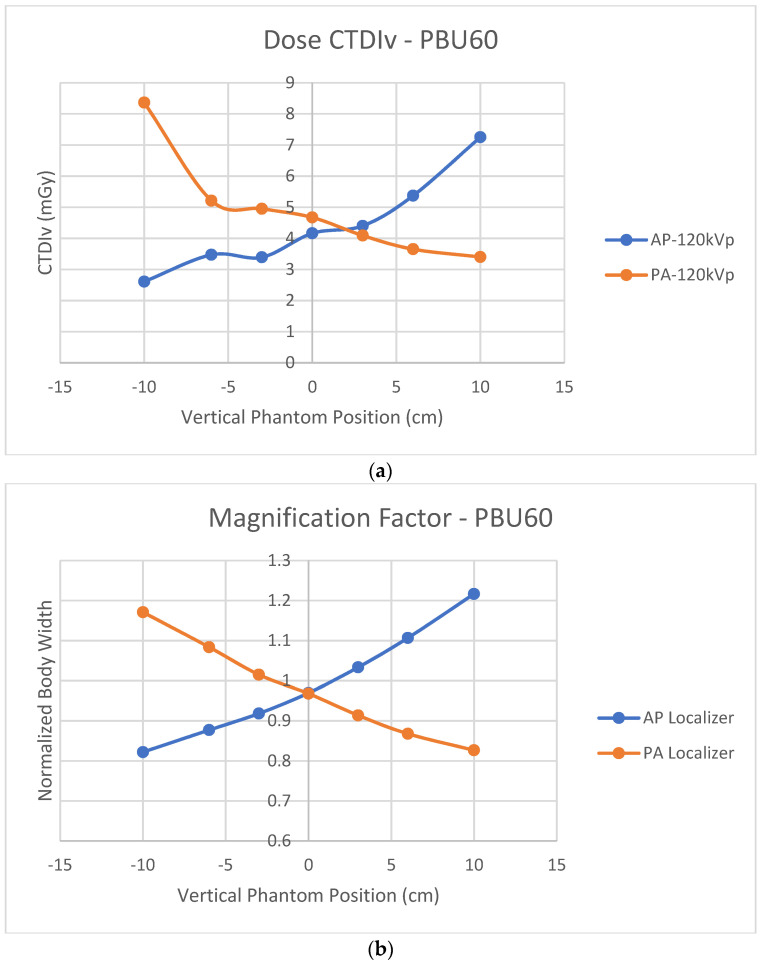
(**a**) Off-center dose index CTDIv variations at 120 kVp using either AP or PA localizers for the PBU60 phantom using the HD750 scanner; (**b**) the magnification factors of the measured body widths to the actual phantom width using either AP or PA localizers for the PBU60 phantom using the HD750 scanner.

Figure 6a shows off-center CTDIv variations using either AP or PA localizers for the PBU60 phantom using the HD750 scanner. Figure 6b shows the normalized body widths to the actual phantom width using either AP or PA localizers for the same PBU60 phantom and HD750 scanner. The cross-over of CTDIv from AP and PA projections is at a point above the iso-center which is consistent with six of the seven CT scanners measured by Paolicchi et al [24]. In contrast, the cross-over of the measured magnification factors from AP and PA projections is at the system’s iso-center. Both AP and PA projection images at the iso-center are minified slightly (normalized body width <1 as shown in Figure 6b).

## 4. Discussion

**Figure 7:**
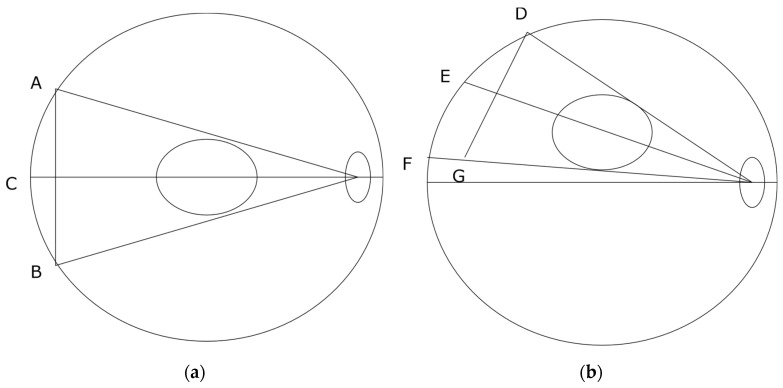
Schematic lateral projections (LAT); (**a**) Phantom is at iso-center position: $\hat{ACB}$ is a curved projection plane and $\bar{AB}$ is a flat projection plane; (**b**) Phantom is at an off-center position: $\hat{DEF}$ is the curved projection plane and $\bar{DG}$ is the flat projection plane.

Figure 2b shows that the measured CTDIv values are almost constant for the APEX scanner as the phantom is lifted above the iso-center. This result is consistent with Philips’ iCT 256 scanners measured by Paolicchi et al. [24]. Merzan et al. [9] stated that a table height correction has been implemented in the Philips’ iCT 256 scanners but not in GE’s Revolution CT scanners; therefore, accurate patient positioning is important. Our results shown in Figure 2b suggest that off-center correction algorithms have been implemented in the APEX system which is in contrast to that of the HD750 scanner. The details of the correction algorithms are not known to us which is proprietary information of the manufacturer. Measuring the size variations of the AP and LAT SPR images from both scanners shows that the difference is in the sizes of the LAT images, which are decreased from the APEX system and increased from the HD750 scanner as shown in Figure 5. The amount of increase or decrease is relatively small for both scanners (±10 mm for a 10 cm off center position). It may be explained by different image projection planes used for the two CT scanners, as shown in Figure 7. If a curved arc (detector) surface image plane $\hat{ACB}$ is used for the SPR as shown in Figure 7a, the length of $\hat{ACB}$ will be increased if the phantom is lifted to an off-center position as $\hat{DEF}$ shown in Figure 7b. However, if a flat image projection plane is used for the SPR ($\bar{AB}$, in Figure 7a), the size of the SPR will be decreased at an off-center position as ($\bar{DG}$) shown in Figure 7b. We believe that the off-center correction algorithms include a change of the SPR image projection planes in the APEX system.

For the HD750 scanner, both CTDIv and CT numbers increased as the size of the SPR images increased. A Pearson correlation coefficient for the CTDIv and SPR image size was calculated to be 0.996 (*p* < 0.01) which suggests a strong correlation between SPR image size and the CTDIv. This is understandable because the tube current is calculated proportional to the estimated body size in ATCM and the CTDIv is proportional to the tube current. When the body is positioned at the iso-center, its size can be estimated using an AP projection assuming an elliptical body shape. When the phantom is lifted above the iso-center, the size of AP SPR image is increased and the ATCM uses an increased size to calculate the tube-current which results in a higher current and a higher CTDIv. The magnification of the body size can be minimized if both AP and LAT projection images are used to estimate the body sizes as recommended by the AAPM [10]. Siemens’ scanners offer an option of using both AP and LAT localizer images in ATCM [9]. It was found that using both localizers would reduce radiation to patients [26]. It is possible that the APEX system uses both AP and LAT localizer images in its correction for off-center positioning. For Siemens CT scanners, we recommend that radiologic technologists use both AP and LAT localizers for the ATCM if this option is available in clinical practice. For GE scanners however, although the option of selecting two localizers such as AP + LAT or LAT + PA is possible, the performance of the ATCM was found to be dependent on the order of the two localizers [15]. We believe that only one localizer (the last one) is actually being used in these non-APEX GE systems for patient size estimation.

For the HD750 scanner, a Pearson correlation coefficient for the CT numbers and the AP-SPR image sizes is calculated to be 0.996 (*p* < 0.00), which is again a strong correlation between CT numbers and the AP-SPR image sizes. For the APEX system, the CT number variations are approximately sinusoidal within a small range of ±2 HU for water as shown in Figure 3b. Dr Hsieh attributed the off-center increase of the CT numbers to the bowtie filter effect [19]. We believe that the off-center variation of CT numbers is a result of the combined effects of the bowtie filter and the estimated body sizes. The size has a stronger effect on CT numbers than that of the bowtie filter in terms of magnitude as evidenced in Figure 3a,b. We believe that the off-center correction in the APEX system is mainly on size effect. The small magnitude sinusoidal variations of the CT numbers (shown in Figure 3b) could be attributed to the bowtie filter effect after corrections for the body size effect in the APEX system.

**Figure 8:**
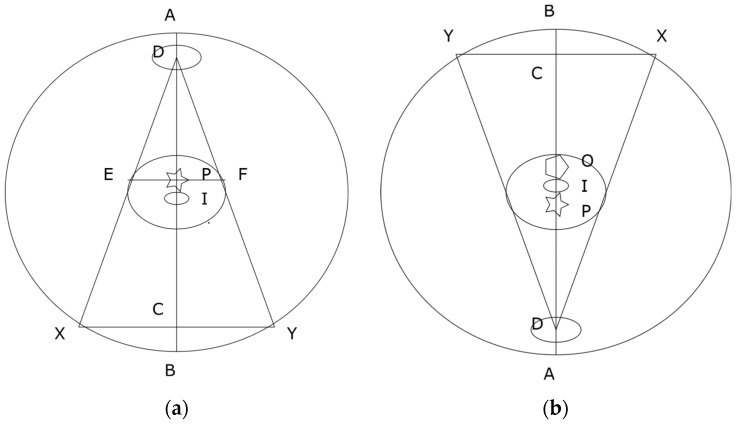
Schematic anterior-posterior (AP) (**a**) and posterior-anterior (PA) (**b**) projections: D is the center of X-ray tube; I is the system’s iso-center; P is the projection center; O is the projection center defined by AP projection; DC is the source to detector distance (SDD) and DI is the source to iso-center distance (SID).

Manufacturers implement ATCM assuming patients are positioned at the iso-center. A single AP, PA or LAT SPR image may be enough to estimate patients’ body size, assuming an elliptical shape for the purpose of ATCM [17,18]. Figure 8a shows a single AP projection. The projection center P is located at the center between the tangential points E and F. The locations E and F are also termed as “grazing” points [27]. The system takes P as the patient’s body center and assumes that body size would be magnified if the body is moved up towards to the tube (D in Figure 8a) and minified if the body is leveled down from the tube. As shown in Figure 8a, the system’s iso-center I or the body’s actual center is below the projection center P. The system’s iso-center I is in a minifying position according to projection center P of Figure 8a. When a PA projection is taken as shown in Figure 8b, the projection center is defined by the AP projection at O. The body’s actual center is again below the pre-defined projection center O but is now in a magnifying position according to O. That explains why the cross-over of the CTDIv is located at a point above the system’s iso-center as shown in Figure 6a. It also explains why using a PA localizer has a higher CTDIv than that of using an AP localizer when patients are positioned at the iso-center [12,13,14,15,16], because the iso-center I is at a magnifying position in the PA projection according to O. This mismatch could be avoided if manufacturers use both AP and PA projections to define the projection center for the ATCM.

At the system’s iso-center, the measured sizes of both AP and PA projection images are smaller than the actual phantom size, as shown in Figure 6b. The reason is that there is a small distance between the system’s iso-center I and X-ray projection center P as shown in Figure 8. The terms of source to iso-center distance (SID) and source to detector distance (SDD) are used in current design of CT scanners [20,28]. The SDD is two times the distance of the source to the projection center P as shown in Figure 8. The distance between P and I is 6.55 cm for GE [20] and 5.00 cm for Siemens [28] CT scanners. The system’s iso-center I is located at a point below the projection center P in AP (Figure 8a) and above the projection center P in PA as shown in Figure 8b. The system’s iso-center I is always in a minifying position for both AP and PA projections. The cross-over of magnification and minification is located at a point above the iso-center I for AP and below the iso-center I for PA projections. If the source to detector distance (SDD) is extended (i.e., C is moved closer to B, or a flat projection plane is used tangentially to the detectors), the projection center P will be moved closer to the system’s iso-center I. Ideally, the cross-over of the magnification and minification should be matched with the system’s iso-center I. The use of a flat projection plane tangential to the detectors for both AP and PA SPR images can reduce the mismatch between the system’s iso-center I and the projection center P. It is recommended that manufacturers use a flat image projection plane tangential to the detectors for both AP and PA SPR images to improve the accuracy of CT imaging.

It is worth noting that the findings of this work are independent of the types of phantoms employed. As shown in Figure 6, the impacts on CTDIv and body-size estimations caused by the mismatch between the projection center and the system’s iso-center were revealed by the measurements using scanner HD750 and PBU60 phantom only. Comparing the measured off-center characteristics of almost constant CTDIv of the APEX system with that of a Philips’ iCT 256 system [24] and the measured decreasing sizes of the LAT SPRs of the APEX system (Figure 5b) led to the conclusion that the off-center correction algorithms are implemented in the APEX system which is in contrast to that of non-APEX GE CT scanners [9]. Contrasting the off-center variation characteristics of the approximately constant CT numbers and dose index CTDIv of the APEX scanner to the linearly increased CT numbers and CTDIv of the HD750 scanner led to the conclusion that the linearly enlarged sizes of SPR images without off-center corrections caused the linear increase of the CT numbers and CTDIv. It suggests that the body size and tube voltage dependent correction scheme [21] should be able to correct for the variations of CT numbers caused by the increased body sizes

## 5. Conclusions

Correction algorithms for patient off-center positioning have been implemented in the Revolution APEX scanner of GE. Possible corrections may include the use of both AP and LAT localizers in the calculations of body sizes and flat projection planes for AP or PA and lateral SPR images. It is recommended that radiologic technologists use both AP and LAT localizers in ATCM if this option is available in Siemens CT scanners. For the vintage HD750 system, the increased CTDIv and CT numbers could be attributed to the enlarged body sizes estimated at off-center positions as well as bowtie filter effects. For the APEX system, a small sinusoidal variation of water CT numbers at the phantom center suggest that the bowtie filter effect remains after correction for the size effect. A mismatch exists between the system’s iso-center and the X-ray projection center in current design of clinical CT scanners. It is recommended that manufacturers use a flat projection plane tangential to the detectors for the AP and PA SPR images to improve the accuracy of body size estimation in ATCM and CT numbers.

## Data Availability

Available from the corresponding author.
